# Statins in conditions other than hypocholesterolemic effects for chronic subdural hematoma therapy, old drug, new tricks?

**DOI:** 10.18632/oncotarget.15092

**Published:** 2017-01-04

**Authors:** Hai Zou, Xing-Xing Zhu, Ya-Hui Ding, Guo-Bing Zhang, Yu Geng, Dong-Sheng Huang

**Affiliations:** ^1^ Department of Cardiology, Zhejiang Provincial People's Hospital, Hangzhou, China; ^2^ Department of Nephrology, Zhejiang Provincial People's Hospital, Hangzhou, China; ^3^ Department of Pharmacy, Zhejiang Provincial People's Hospital, Hangzhou, China; ^4^ Department of Neurology, Zhejiang Provincial People's Hospital, Hangzhou, China; ^5^ Department of Hepatobiliary Surgery, Zhejiang Provincial People's Hospital, Hangzhou, China

**Keywords:** statins, chronic subdural hematoma, endothelial progenitor cells, vascular endothelial growth factor, inflammation

## Abstract

Chronic subdural hematoma (CSDH) is one of the most common intracranial hematomas worldwide with a high incidence in the general population. However, the optimum treatment for CSDH is Burr-hole drainage with or without rinse Considering the poor outcomes of CSDH in aged patients, and ambiguous prediction of recurrence in many sides of recurrent CSDHs who have been analyzed, new effective therapies are needed for those CSDHs who are predicated to have poor prognosis for surgery and/or have a higher risk of recurrence. Statins, which is the first-line treatment for patients with high cholesterol and coronary heart disease. However, statins are still not solely limited in the treatment of these diseases. It has been demonstrated that statins could improve CSDH due to its effect of regulation of angiogenesis and inflammation. In this review, in order to provide potential new treatment for CSDH we summarize the recent findings of statins in CSDH in order to try to clarify the mechanisms of this effect.

## INTRODUCTION

Chronic subdural hematoma (CSDH) is one of the most common intracranial hematomas worldwide with an incidence of 58 per 10,000 among people 70 years or older and 5 per 10,000 people in the general population [1]. Standard treatment for CSDH is Burr-hole drainage with or without rinsing [2]. However, despite surgical treatment, recurrence rates among treated patients are still relatively high. What's more, as most CSDH patients are elderly, they are at high risk of suffering from pneumonia, peri-operational infection and high-surface-tension pulmonary edema following surgery [3]. Considering the poor outcomes of aged patients with CSDH and the likelihood of recurrence, new effective therapies are needed for patients with CSDHs that have a poor prognosis for surgery and/or at high risk for recurrence.

Despite the controversy and ineffectiveness of current therapies and the need for new therapeutic strategies, little is known about the mechanisms underlying CSDH progression [4]. Increasing evidence indicate that impaired angiogenesis in the neomembrane and localized inflammation may play an important role in CSDH formation. Impaired angiogenesis may induce blood leakage from immature vessels of the neomembrane. Repair of vessel leakage is further impeded by localized inflammation [5–8].

Recent studies support a potential therapeutic role for Statins, 3-hydroxy-3-methylglutaryl coenzyme A (HMG-CoA) reductase inhibitors approved for cholesterol reduction, in enhancing angiogenesis and reducing inflammation associated with CSDH [9, 10]. The statin atorvastatin was shown to improve angiogenesis and increases the level of circulating endothelial progenitor cells (EPCs)—cells essential to new blood vessel formation—by activating the endothelial nitric oxide synthase (eNOS), endothelial protein kinase Akt/PKB and Notch1 in patients with CSDH [11–14]. Atorvastatin also inhibited inflammation and decreased the levels of pro-inflammatory molecules [15–17]. A better understanding of the mechanisms behind these therapeutic statin effects may provide novel targets for future therapeutics for CSDH. In this review, we summarize recent findings regarding the mechanism and role of statins in CSDH.

### Statins mediate endothelium renewal and repair via endothelial progenitor cells

Endothelial progenitor cells (EPCs) are bone marrow-derived late stage stem cells that circulate in the blood. EPCs promote vascular repair of denuded vessel walls and renew endothelium [18] through various processes including mobilization and differentiation [19]. Many factors such as trauma and hematomas mobilize EPCs from the bone into the peripheral bloodstream [20]. These mobile EPCs home to damaged tissue and differentiate into mature endothelial cells that promote vascular repair [21–23].

Song et al. found that the level of peripheral blood EPCs in patients with CSDH was significantly lower than in healthy patients, and that the postoperation EPC level for recurrent patients was significantly lower than in non-recurrent patients. These data suggest that low EPC levels impair endothelium repair capacity, and thus increase the risk of CSDH development and recurrence [19].

A series of studies have demonstrated that statins promote EPC processes involved in endothelium renewal and repair and reduce senescence and apoptosis. In Landmesser's 2004 study, they showed that increased endothelial nitric oxide (eNO) availability was required for statin-induced improvement of EPC mobilization [24]. Liu et al. showed that atorvastatin blocked miR-221 and miR-222 inhibition of cell migration, tube formation and wound healing by endothelial cells *in vitro* [25]. In addition, in coronary artery disease patients, miR-221 and miR-222 expression was inversely related to EPC levels [26]. There is also evidence that statins induce mobilization and migration of EPCs through activation of matrix metalloproteinase-2 (MMP-2) and -9 (MMP-9), two proteins involved in extracellular matrix (ECM) degradation [27].

Statin promotes EPC activity through multiple pathways. In an ischemic hind limb C57BL/6 mouse model, statins enhanced EPC proliferation and migration and decreased apoptosis through activation of the Akt/eNOS pathway, effects that were inhibited by the PI3K/Akt inhibitor, LY294002 [27]. Statin treatment also decreased EPC apoptosis by activation of the Akt/NOS pathway [27]. In a study conducted by Vasa et al, statin treatment activated the Akt/ eNOS pathway and mediated vascular endothelial growth factor (VEGF)-induced EC migration [28]. Statins have also been shown to enhance migration and attenuate cellular senescence by upregulating telomere repeat-binding factor (TRF2) [29]. Statin simvastatin may also promote EPC adhesion by up-regulating integrin α5 and β1, subunits of the fibronectin receptor [18]. What's more, a recent study showed that, in cultured human ECs, low-dose atorvastatin inhibited EC senescence by suppressing reactive oxygen species (ROS) production [30].

Interestingly, statin-mediated effects are dose dependent. Low doses of statins promoted angiogenesis[10, 28], while high doses of statins inhibited the growth and migration of EPCs [31, 32].

The endothelial promoting effects of statins have revealed the cholesterol lowering therapy to be a potentially novel therapeutic approach to treating CSDH. More researches are needed to further illustrate the functional mechanisms of statins in CSDH and determine the potential clinical uses for statins for CSDH treatment.

### Statins improve vessel maturation by inhibiting vascular endothelial growth factor expression

Vascular endothelial growth factors (VEGFs) and their receptors play critical roles in vasculogenesis, vascular permeability and angiogenesis [33, 34]. VEGF is also highly expressed in the serum of patients with CSDH and in both the dura and outer membrane of the CSH [6, 35]. This high VEGF concentration in the hematoma fluid may be of major pathophysiological importance in the generation and steady increase of the hematoma volume [36]. Interestingly, VEGF promotes angiogenesis, but when expressed persistently at a high level, it also inhibits the maturation of new vessels [37]. This is observed in subdural hematomas. Excessive expression of VEGF increases the number of blood vessels with high permeability [38], but also leads to immature and unstable vessels [39].

A meta-analysis of randomized controlled trials showed that statin treatment was associated with a significant reduction in circulating VEGF concentrations, particularly lipophilic statins and statins administered for over long periods of time [40]. Thus inhibiting VEGF may be beneficial for treating patients with CSDHs in the early stages.

### Statins improve functional recovery and vessel maturation by inhibiting inflammation in CSDH

There is increasing evidence showing that localized inflammation plays a key role in the formation of a CSDH [41–43]. A similar imbalance between anti- and pro-inflammatory molecules has been found in the CSDH fluid and this imbalance could influence the vascular permeability of the hematoma neomembrane and angiogenesis [7, 8, 44, 45]. Recent reports revealed that inflammatory factors, such as TNF-α, IL-6 and IL-10 are highly expressed in the hematoma fluid of patients with CSDH [44, 46].. The inflammatory reaction alters osmotic pressure of hematoma liquid, and may impact angiogenesis by altering vessel maturation [47].

Several anti-inflammatory therapies are being explored for the treatment of CSDH, including glucocorticoids, mannitol and angiotensin-converting enzyme inhibitors [3] [48–50]. However, these drugs are associated with adverse effects. For example, while dexamethasone, a potent anti-inflammatory drug, successfully cures CSDH [50], it is associated with many side effects such as also cause increased weight, thrush, nausea, agitation, acne, which seriously influenced its clinical use [51].

Statins have also been shown to suppress inflammation and decrease pro-inflammatory molecule levels in CSDH. Li et al. has found that rats with subdermal hematomas (SDHs) treated by atorvastatin experienced better behavioral function recovery and rapid volume elimination, had more new CD31+ blood vessels and less neutrophilic granulocytes than rats treated with saline [52]. Atorvastatin treatment also significantly decreased TNF-α and IL-6 levels [52]. These studies suggest that statin-induced inflammation modulation significantly influences SDH elimination and functional recovery in rats [52], and may be beneficial in treating CSDH. More clinical research is needed to confirm the role and the mechanisms that inflammation plays in CSDH.

## CONCLUSIONS

**Figure 1:**
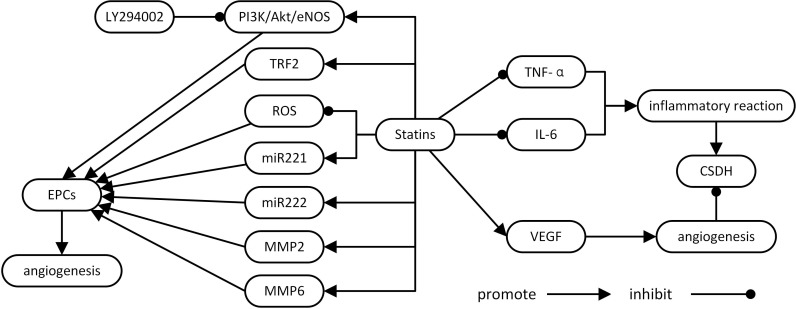
Potential mechanisms and the role of statins in CSDH CSDH: chronic subdural hematoma; EPCs: endothelial progenitor cells; IL-6: interleukin-6; PI3K/Akt/eNOS: phosphatidylinositol 3-kinase/ protein kinase B/ endothelial nitric oxide synthase; ROS: reactive oxygen species; TRF2: TTAGGG repeat binding factor-2; TNF-α: tumor necrosis factor -α; VEGF: vascular endothelial growth factor.

Statins have pleiotropic effects including anti-inflammation, neuroprotection and angiogenesis regulation [12, 15, 53], and may provide a novel treatment method for CSDH. In this review, we show that statins may impact CSDH development and progression by inhibiting inflammation and improving blood vessel formation and maturation. Evidence from a series of studies showed that statins exhibit ameliorative effects by promoting EPC mobilization and activation, suppressing VEGF and inhibiting inflammation (Figure 1).

ECPs are a key factor in hemostasis and vascular repair through mobilization, tendency, adhesion, proliferation, and differentiation [19]. Statins promote EPC mobilization by increasing eNOS and activation of MMP-2 and -9 [24], enhance EPCs migration by inhibiting miR- 221 and miR-222 [25, 26], enhance EPC proliferation by activating the Akt/eNOS pathway[27, 28] and enhance migratory capacity and attenuate cellular senescence by up-regulating TRF2 [29]. We speculate that statins may improve CSDH via these mechanisms, but more research is still needed to validate these mechanisms and investigate these therapeutic effects.

VEGF is another major component in CSDH maintenance. VEGF expression is markedly up-regulated in both the dura and outer membrane of CSDH [35, 36]. Treatment with statins reduces VEGF expression. Thus, treatment with statins in the early stages of a CSDH may prevent VEGF upregulation and further progression of the hematoma [52].

Localized inflammation reaction and inflammatory factors, such as tumor necrosis factor-α (TNF-α), interleukin-6 (IL-6) and IL-10, also contribute to CSDH formation [41–43, 46]. Statin-induced inflammatory modulation significantly improved functional recover, SDH elimination, and vessel maturation in rats with CSDH Treatment also significantly decreased TNF-α and IL-6 expression [52].

In conclusion, statins are a promising therapeutic intervention for the treatment of CSDH. More research is needed to further illustrate the functional mechanisms of statins in CSDH.

## EXPERT OPINION

In this report, we have discussed the therapeutic potential of statins in treating CSDH based on studies conducted in the last five years. We have discovered several interesting findings on how statins may influence CSDH. The first one being that different doses of stains have different effects on EPCs and VEGF. Low doses of statins promote angiogenesis [10, 28], but high doses inhibit EPC growth and migration which inhibits angiogenesis [31, 32]. Interestingly, VEGF promotes angiogenesis, but when expressed at a high level it also inhibits the maturation of new vessels [37]. These doses-dependent effects need further investigation.

The second interesting finding is that stains may have varying effects via VEGF in different stages of CSDH. Li et al. found that VEGF expression was high and then slowly decreased following treatment with a statin [52]. We speculate that, in early CSDH, inhibiting VEGF may be beneficial for preventing further CSDH development.

While an increasing number of studies are exploring the role of statins in CSDH, it is important that the biological and underlying mechanisms of its effects remain a major focus of future studies.

## FIVE-YEAR VIEW

A randomized clinical trial about the effect and safety of atorvastatin to treat CSDH in ClinicTrials.gov (NCT0236232) has been completed in October, 2016. Excitedly, the result of atorvastatin on CSDH was positive in this study. We are waiting for the publication of this important article. Over the next 5 years, we expect statins to be a strong contender as a therapeutic for CSDH. Pharmacological evidence and clinical trial results support the interpretation that statins treat CSDHs by regulating potential signaling pathways, such as Akt/eNOS pathway, MMP-2 and MMP-9, ROS, TNF-α and IL-6.We predict that statins will be approved for the treatment of these CSDHs. Further research is need to confirm the mechanism, therapeutic effects and safety of statin use in treating CSDH.

### Key points

- Different doses of stains have different effects on EPCs and VEGF. Low dose of statins promoted angiogenesis, but high dose of statins inhibited the growth and migration of EPCs to inhibit angiogenesis.
- Stains may has different role via VEGF in different stage of CSDH. In early CSDH, inhibiting VEGF may be benefit for the occurrence of CSDH, but in late CSDH, increasing VEGF may be benefit for the occurrence of CSDH.
